# The effect of uveitis and undiagnosed spondyloarthritis: a systematic review and meta-analysis

**DOI:** 10.1038/s41598-023-41971-z

**Published:** 2023-09-07

**Authors:** Shih-Ching Lee, Chung-Han Yang, Yun-Chen Tsai, Kuang-Hui Yu

**Affiliations:** 1 Division of Rheumatology, Allergy and Immunology, Department of Internal Medicine, Chang Gung Memorial Hospital, No. 5, Fu-Shin St., Kuei-Shan, 333 Tao-Yuan, Taiwan; 2 Chang Gung University, Tao-Yuan, Taiwan; 3 Graduate Institute of Biomedical Electronics and Bioinformatics, National Taiwan University, Taipei, Taiwan; 4 Graduate Institute of Clinical Medicine, National Taiwan University, Taipei, Taiwan

**Keywords:** Immunology, Rheumatology

## Abstract

Delay diagnosis of spondyloarthritis (SpA) is associated with poor functional ability and quality of life. Uveitis is the most frequent extraarticular manifestation in SpA, and its prevalence increases with longer disease duration. This study examines the effect of uveitis on the disease activity and functional outcome of undiagnosed SpA. We reviewed published and unpublished studies. Data were pooled using the random-effects model; pooled means, and mean differences (MDs) were calculated. In the included 14 studies, disease activity, functional index, and inflammatory markers were measured in 2581 patients with SpA with uveitis and 13,972 without. The pooled mean delay in diagnosis of SpA with uveitis (6.08 years; 95% CI 4.77 to 7.38) was longer than those without (5.41 years; 95% CI 3.94 to 6.89). The Bath Ankylosing Spondylitis Disease Activity Index (BASDAI) score was the highest for a delay of 2–5 years (5.60, 95% CI 5.47 to 5.73) and the Bath Ankylosing Spondylitis Functional Index (BASFI) score was the lowest for a delay of < 2 years (2.92, 95% CI 2.48 to 3.37) and gradually increased to delay of > 10 years (4.17, 95% CI 2.93 to 5.41). Patients with SpA with uveitis had higher trend of Ankylosing Spondylitis Disease Activity Score (ASDAS)-CRP and BASDAI. The delay to diagnosis was longer in SpA with uveitis, and disease activity was often higher than those without uveitis. Early diagnosis of SpA with timely initiation of an appropriate management plan may reduce the adverse effects of the disease and improve functional ability.

## Introduction

Spondyloarthritis (SpA) is a chronic inflammatory disease that predominantly manifests in the spine with an insidious onset involving deep, dull pain in the lower back. SpA involves spinal and extraspinal signs and symptoms^1^. For clinical purposes, five disease subtypes of SpA are recognized: ankylosing spondylitis (AS), psoriatic spondyloarthritis (PsA), reactive arthritis, SpA associated with inflammatory bowel disease, and undifferentiated SpA^2^. Moreover, SpA can result in peripheral arthritis, enthesitis, dactylitis, and other extraarticular manifestations (EAMs), with uveitis being the most commonly (observed in 25–30% of patients)^3,4^.

The diagnosis of SpA is often considerably delayed, and early diagnosis and intervention can slow down the development and progression of structural changes^4^. The pathological basis for SpA-related inflammation is recurrent mechanical stress triggering the tissue microdamage and repair processes that occur exactly at the same target sites such as the anterior uveal tract, aortic root and valve, lung apex, and enthesis organ structures^5^. Several studies have examined the prevalence of SpA with uveitis, and a longer disease duration and a higher proportion of human leukocyte antigen B27 (HLA-B27) were noted^3,4,6–10^. Delay of diagnosis is longer in SpA than in many other rheumatic diseases^11^. Prolonged delay was associated with poor outcomes including functional impairment and decreased quality of life^11^. Uveitis may be diagnosed before SpA or even before the occurrence of SpA symptoms^12^. As the most common EAM of SpA, uveitis may be the earliest or first symptom of SpA^12^ and thus may provide an opportunity for early SpA recognition. The Dublin Uveitis Evaluation Tool (DUET) assessment algorithm can aid in the early diagnosis of SpA in patients with uveitis, boasting a sensitivity of 95% and a specificity of 98%^13^. However, studies examining the effect of uveitis on the disease activity and functional outcome of SpA have reported inconsistent results^6,10,14,15^.

In clinical practice, the functional ability of patients with SpA is evaluated using the Bath Ankylosing Spondylitis Functional Index (BASFI). Moreover, disease activity is quantified using two evaluation tools, namely the Bath Ankylosing Spondylitis Disease Activity Index (BASDAI) and Ankylosing Spondylitis Disease Activity Score (ASDAS)^16^. The BASDAI contains only subjective clinical items, whereas the ASDAS contains both subjective clinical items and objective laboratory measures including the erythrocyte sedimentation rate (ESR) and the C-reactive protein (CRP) level^1^.

Due to inconclusive emerging data regarding the disease activity and functional ability of patients with SpA with and without uveitis^7,9,15,17–21^, this study aimed to provide further insight by conducting a meta-analysis of existing studies. Specifically, the study examined the disease activity and functional outcome of SpA, while also analyzing differences between patients with SpA with and without uveitis.

## Results

### Characteristics of the study population

**Figure 1:**
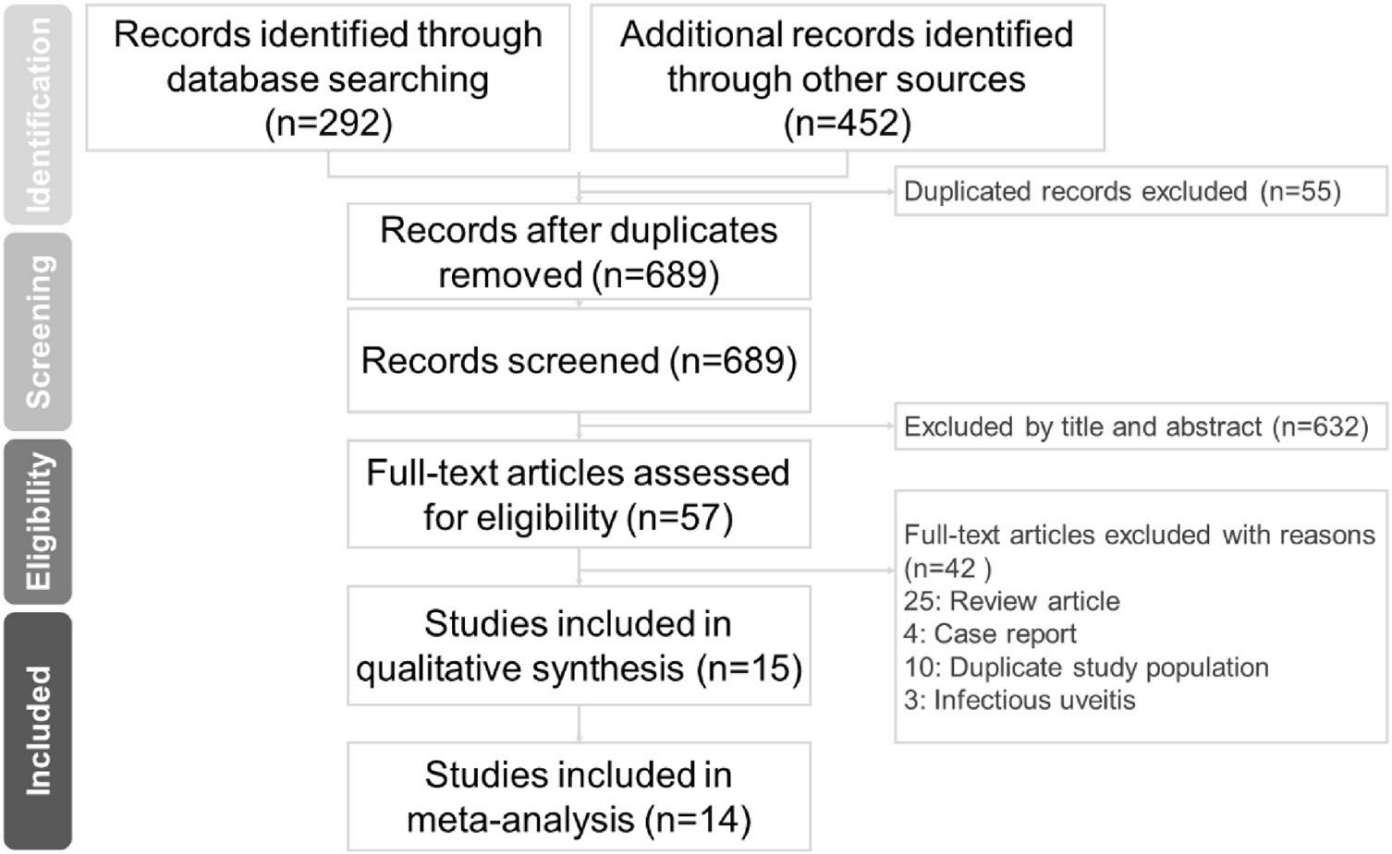
PRISMA flow diagram for studies retrieved through the electronic search and the selection processes.

In the present study, we identified 689 articles from the online databases, among which 632 irrelevant articles were excluded. The remaining 57 articles were assessed for eligibility. Finally, 11 articles^6–10,14,15,17–19,21^ and 3 conference proceedings^20,22,23^ including 16,553 patients with SpA were included in the meta-analysis. The PRISMA flow diagram (Fig. 1) for studies retrieved through the electronic search and the selection process for study inclusion. The quality of the included studies was assessed using the RoBANS tool^24^ (Supplementary Fig. S1). In particular, the studies included 2581 patients with SpA with uveitis and 13,972 patients with SpA without uveitis. Table 1 lists the detailed characteristics of each study. Among the included studies, three were conducted in Asian populations, including two studies in China^9,10^ and one study in Taiwan^17^. Another 11 studies examined Caucasian populations from Europe, Latin America, and Turkey^6–8,14,15,18–23^ (Table 1).

**Table 1:** Characteristics of the included studies.

Study	Characteristics	Criteria	Patient (n)*	% male	Delay (y)*	Duration (y)*	HLA-B27 (+) %*
Chen 2007^17^	Taiwan, cross-sectional, AS	NY	23/123	70.5	9.6/8.7	9.7/8.7	100/87
Wendling 2012^6^	French, cohort, SPA	ESSG	60/648	46.3	1.9/1.6	NA	63.3/57.1
Gehlen 2012^18^	Brazil, cross- sectional, SPA	ESSG	22/80	58.8	17.9/13.1	NA	66.6/69.0
Sampaio-Barros 2013^19^	RESPONDIA, cohort, SPA	ESSG	372/1640	NA	7.9/5.9	NA	72.1/60.6
Berg 2014^7^	Norway, cross-sectional, AS	NY	84/75	61.6	NA	26.8/21.4	75/68
Diss 2014^20^	UK, cohort, SPA, AS, PsA	ESSG, NY, CASPAR	24/43	37.5	NA	15.5/11.3	100/100
Costa 2015^21^	Brazil, cohort, SPA	ESSG	285/1207	72.3	NA	NA	NA
Lian 2015^10^	China, cohort, SPA	ASAS	182/854	87.7	NA	5.1/5.7	91.8/94.0
Essers 2015^14^	OASIS, cohort, AS	NY	39/177	71.3	NA	25.9/19.3	89.7/83.1
Przepiera-Bedzak 2016^8^	Poland, cohort AS, PsA, SAPHO	NY, CASPAR, Kahn	35/252	54.7	NA	11.3/5.3	94.4/47.1
Kasifoglu 2018^22^	Turkey, cohort, SPA	NA	269/2359	56.8	3.0/2.0	10.7/6.7	69.2/49.5
Bilge 2019^23^	Turkey, cohort, SPA	NA	491/4066	56.1	3.1/2.0	11.2/7.4	70.5/49.3
Redeker 2020^15^	Germany, cohort, SPA	NA	463/1266	53.8	5.6/5.6	29.8/23.5	90.4/84.6
Man 2021^9^	China, cohort, SPA	NY	232/1182	NA	9.9/7.6	9.9/7.8	87.8/83.3

*Delay* delay time of diagnosis, *NA* not available, *NY* 1984 modified New York criteria for ankylosing spondylitis, *ESSG* European Spondylarthropathy Study Group criteria for SPA, *ASAS* Assessment of Spondylarthritis International Society classification Criteria for SPA, *CASPAR* Classification Criteria for Psoriatic Arthritis, *Kahn* Classification Criteria for SAPHO syndrome, *AS* Ankylosing Spondylitis, *SPA* Spondylarthritis, *PsA* Psoriatic Arthritis, *SAPHO syndrome* synovitis acne pustulosis hyperostosis osteitis syndrome.

*Uveitis (+)/Uveitis (−).

Eleven studies presented BASDAI and BASFI results^6,7,10,14,15,17–20,22,23^, whereas two studies examined only the BASDAI in the patient population^8,21^. Five studies reported ASDAS results with ESR and CRP levels^6–8,10,14^, and one study examined the ASDAS only with CRP^9^. Three studies^19,22,23^ reported ESR and CRP levels without evaluating the ASDAS (Table 2).

**Table 2:** Comparison of clinical data in spondylarthritis patients with and without uveitis of the Included studies.

Study	BASDAI		BASFI		ASDAS-CRP		CRP		ESR	
	Uveitis		Uveitis		Uveitis		Uveitis		Uveitis	
	+ yes	− no	+	−	+	−	+	−	+	−
Chen 2007^17^	4.9 (2.4)	3.9 (2.0)	4.2 (2.9)	2.8 (2.4)						
Wendling 2012^6^	4.3 (2.1)	4.5 (2.0)	2.6 (2.2)	3.1 (2.3)	2.6 (1.0)	2.5 (1.0)	9.2 (12.2)	9.0 (14.6)	11.8 (13.0)	14.0 (15.1)
Gehlen 2012^18^	4.4 (2.4)	3.3 (2.5)	3.4 (3.3)	4.7 (3.0)						
Sampaio-Barros 2013^19^	4.3 (2.4)	4.3 (4.5)	4.5 (2.9)	4.2 (2.9)			11.4 (18.3))	8.0 (14.5)	25.3 (21.2)	24.0 (19.8))
Berg 2014^7^	4.0 (2.0)	3.5 (1.7)	2.7 (2.2)	1.7 (1.7)	2.4 (1.0)	2.2 (0.9)	5.0 (5.2)	5.0 (7.4)	17.0 (14.8)	17.0 (16.3)
Diss 2014^20^	5.9 (0.4)	5.8 (0.3)	4.5 (0.6)	4.5 (0.4)						
Costa 2015^21^	4.2 (2.5)	4.2 (2.6)								
Lian 2015^10^	6.6 (2.8)	5.9 (3.1)	5.7 (2.6)	6.5 (3.7)	2.4 (0.6)	2.2 (0.5)	38.4 (17.9)	40.3 (19.3)	78.6 (24.5)	92.3 (33.1)
					2.5 (0.7)*	2.8 (0.9)*				
Essers 2015^14^	3.4 (2)	3.5 (2.2)	3.3 (2.3)	3.4 (2.7)	2.7 (0.8)	2.7 (1.1)	15.4 (15.1)	18.6 (26.2)	11.7 (7.9)	15.3 (17.0)
Przepiera-Bedzak 2016^8^	5.8 (2.8)	3.9 (2.7)			2.8 (0.9)*	2.4 (0.9)*	11.4 (13.7)	6.1 (7.0)	18.0 (22.2)	14.7 (13.7)
Kasifoglu 2018^22^	5.6 (2.2)	5.7 (2.1)	3.6 (2.4)	4.3 (2.7)			17.9 (20.7)	14.8 (17.0)	29 (27.4)	24.7 (21.5)
Bilge 2019^23^	5.3 (2.3)	5.6 (2.1)	3.6 (2.4)	4.3 (2.7)			17.5 (21.4)	14.7 (17.0)	27.7 (26.0)	23.3 (20.7)
Redeker 2020^15^	4.4 (0.1)	4.5 (0.1)	4.2 (0.1)	4.0 (0.1)						
Man 2021^9^					2.2 (1.0)	2.0 (1.0)				

Values are shown as mean (SD).

*BASDAI* Bath Ankylosing Spondylitis Disease Activity Index, *BASFI* Bath Ankylosing Spondylitis Functional Index, *ASDAS* Ankylosing Spondylitis Disease Activity Score, *CRP* C-reactive protein, *ESR* erythrocyte sedimentation rate.

*ASDAS-ESR.

### Statistical pooling of outcomes and meta-analysis

The pooled mean values of the BASDAI and BASFI for delay in the diagnosis (the time from the onset of symptoms to the diagnosis) of SpA (including with and without uveitis patients) were grouped into the following periods: ≤ 2, 2–5, 5–10, and > 10 years. The pooled mean values of the BASDAI and BASFI were 4.46 (95% CI 4.32 to 4.61) and 2.92 (95% CI 2.48 to 3.37), respectively, for ≤ 2 years; 5.60 (95% CI 5.47 to 5.73) and 3.98 (95% CI 3.67 to 4.28), respectively, for 2–5 years; 4.40 (95% CI 4.32 to 4.49) and 4.06 (95% CI 3.91 to 4.20), respectively, for 5–10 years; and 3.74 (95% CI 2.63 to 4.85) and 4.17 (95% CI 2.93 to 5.41), respectively, for > 10 years (Table 3).

**Table 3:** The pooled mean and mean difference of clinical data in spondyloarthritis patients with and without uveitis of the included studies.

Subgroup	Uveitis (+)		Uveitis (–)		Mean difference	95% CI	*p* value (I^2^)
	Pooled mean	95% CI	Pooled mean	95% CI			
Delay diagnosis (y)	6.08	4.77 to 7.38	5.41	3.94 to 6.89	1.04	0.28 to 1.80	0.008 (83%)
ASDAS	2.47	2.31 to 2.64	2.32	2.15 to 2.50	0.18	0.11 to 0.25	< 0.001 (0%)
BASDAI	4.87	4.41 to 5.32	4.53	4.11 to 4.96	0.11	− 0.06 to 0.28	0.21 (76%)
BASFI	3.89	3.53 to 4.25	3.97	3.65 to 4.28	− 0.12	− 0.46 to 0.21	0.47 (92%)
CRP (mg/L)	15.78	8.38 to 23.18	14.53	8.69 to 20.38	1.48	− 0.17 to 3.14	0.08 (64%)
ESR (mm/h)	28.19	7.64 to 48.75	28.16	17.91 to 38.42	− 0.80	− 4.62 to 3.03	0.68 (90%)
ASDAS-CRP	2.42	2.26 to 2.59	2.31	2.11 to 2.51	0.17	0.10 to 0.24	< 0.001 (0%)
ASDAS-ESR	2.76	2.47 to 3.05	2.37	2.26 to 2.48	0.03	− 0.65 to 0.72	0.92 (94%)

Subgroup	BASDAI		BASFI		Study number		
	Pooled mean	95% CI	Pooled mean	95% CI			
≤ 2 years	4.46	4.32 to 4.61	2.92	2.48 to 3.37	1 (6)		
> 2 years, ≤ 5 years	5.60	5.47 to 5.73	3.98	3.67 to 4.28	2 (22, 23)		
> 5 years, ≤ 10 years	4.40	4.32 to 4.49	4.06	3.91 to 4.20	3 (15, 17, 19)		
> 10 years	3.74	2.63 to 4.85	4.17	2.93 to 5.41	1 (18)		

**Figure 2:**
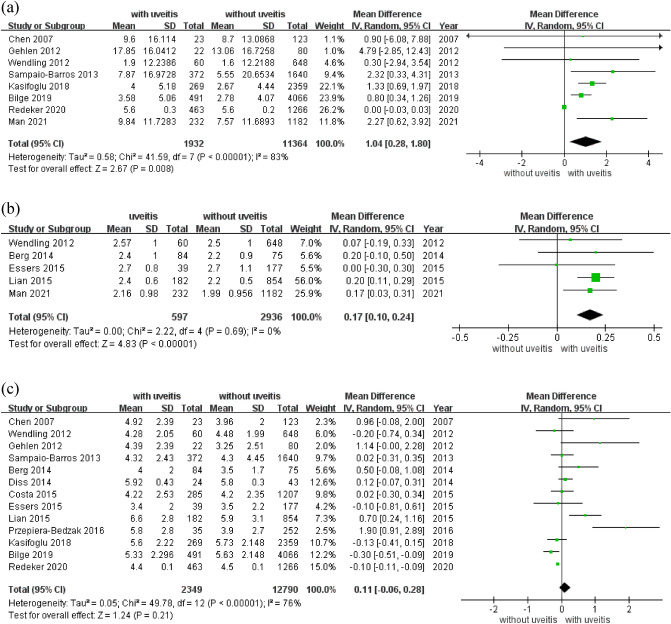
Forest plot of the mean difference of delay diagnosis, ASDAS-CRP, and BASDAI in patients of spondyloarthritis with and without uveitis. (**a**) delay diagnosis, (**b**) ASDAS-CRP, (**c**) BASDAI.

The pooled mean values of delay in the diagnosis of SpA with and without uveitis were 6.08 (95% CI 4.77 to 7.38) years and 5.41 (95% CI 3.94 to 6.89) years, respectively. For continuous variables, mean differences (MDs) and 95% confidence intervals (CIs) were calculated. The delay in diagnosis was significantly longer in patients with uveitis than in those without uveitis (MD 1.04; 95% CI 0.28 to 1.80, *p* = 0.008; Table 3; Fig. 2).

The pooled mean ASDASs were 2.47 (95% CI 2.31 to 2.64) and 2.32 (95% CI 2.15 to 2.50) for patients with and without uveitis, respectively (Table 3; Supplementary Fig. S2). The disease activity was significantly higher in patients with uveitis than in those without uveitis (MD 0.18; 95% CI 0.11 to 0.25, *p* < 0.001, I^2^ = 0%; Table 3; Supplementary Fig. S3). Six^6–10, 13^ of the 14 studies examined the ASDAS, among which four examined the ASDAS-CRP, one^10^ evaluated both ASDAS-CRP and ASDAS-ESR, and one^8^ examined only ASDAS-ESR. The ASDAS-CRP score was significantly higher in patients with SpA with uveitis (MD 0.17; 95% CI 0.10 to 0.24, *p* < 0.001, I^2^ = 0%; Table 3; Fig. 2). No significant difference in the ASDAS-ESR score was observed between patients with SpA with and without uveitis (MD, 0.03; 95% CI − 0.65 to 0.72, *p* = 0.92; Table 3; Supplementary Fig. S3).

The pooled mean BASDAI scores were 4.87 (95% CI 4.41 to 5.32) and 4.53 (95% CI 4.11 to 4.96) in patients with and without uveitis, respectively (Table 3; Supplementary Fig. S2). BASDAI score showed a trend of high in disease activity was observed between patients with and without uveitis (MD 0.11; 95% CI − 0.06 to 0.28, *p* = 0.21, I^2^ = 76%; Table 3; Fig. 2). The pooled mean BASFI scores were 3.89 (95% CI 3.53 to 4.25) and 3.97 (95% CI 3.65 to 4.28) for patients with and without uveitis, respectively (Table 3; Supplementary Fig. S2). No significant difference in BASFI scores was observed between patients with and without uveitis (MD − 0.12, 95% CI − 0.46 to 0.21, *p* = 0.47; Table 3; Supplementary Fig. S3).

The pooled mean CRP levels were 15.78 (95% CI 8.38 to 23.18) mg/L and 14.53 (95% CI 8.69 to 20.38) mg/L in patients with and without uveitis, respectively (Table 3; Supplementary Fig. S2). The CRP level was non-significantly higher in patients with uveitis than in those without uveitis (MD 1.48; 95% CI − 0.17 to 3.14 mg/L, *p* = 0.08; Table 3; Supplementary Fig. S3). The pooled mean ESR levels were 28.19 (95% CI 7.64 to 48.75) mm/h and 28.16 (95% CI 17.91 to 38.42) mm/h in patients with and without uveitis, respectively (Table 3; Supplementary Fig. S2). No significant difference in the ESR level was observed between patients with and without uveitis (MD − 0.80; 95% CI − 4.62 to 3.03 mm/h, *p* = 0.68; Table 3; Supplementary Fig. S3).

### Subgroup analysis

The heterogeneity among the included studies was obvious and we performed subgroup analysis for the mean difference of BASDAI score observed between SpA patients with and without uveitis. In the included studies, there were three study characteristics that may have affected the results. First, the delay diagnosis varied from 1.6 to 17.9 years. Second, the characteristics of participants varied in diagnostic criteria of SpA, sample size of patients and gender ratios. Third, the study design varied between cross-sectional and cohort studies.

**Figure 3:**
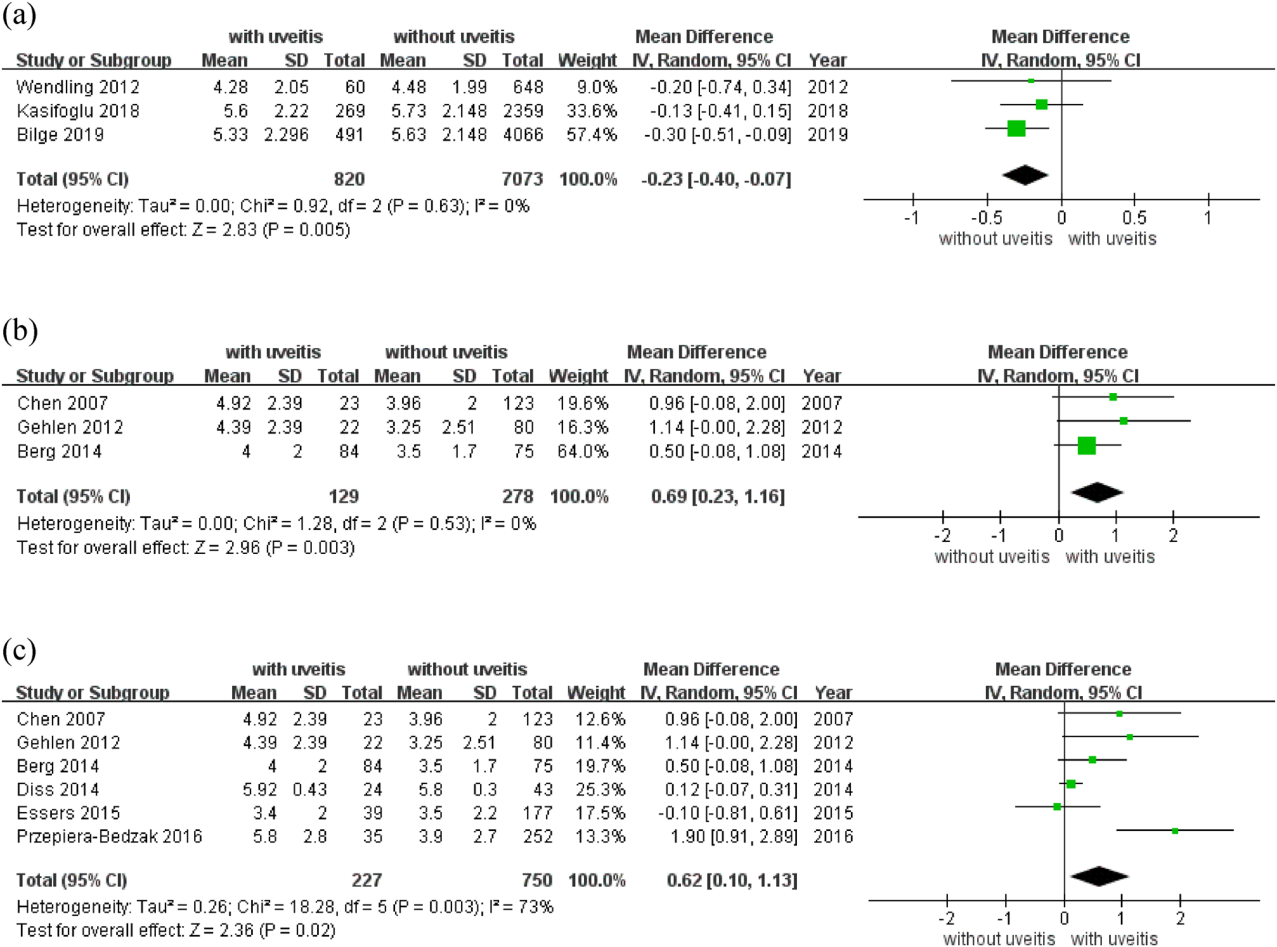
Forest plot of the subgroup analysis of BASDAI in patients of spondyloarthritis with and without uveitis. (**a**) Delay diagnosis less than 5 years, (**b**) Study Design: Cross-sectional, (**c**) Sample Size <500 patients.

In the subgroup analysis, the BASDAI score was significantly low in patients of SpA with uveitis and delay diagnosis less than 5 years (MD − 0.23; 95% CI − 0.40 to − 0.07, p = 0.005, I^2^ = 0%) and it was high in study design as cross-sectional method (MD 0.69; 95% CI 0.23 to 1.16, p = 0.003, I^2^ = 0%; Table 4; Fig. 3). This could be due to the disease activity of SpA with uveitis is higher than without. However, if diagnosed and treated within the first five years from symptom onset, the disease activity of SpA with uveitis can be lower than without. The non-significant result and heterogeneity was found in study design as cohort method (MD 0.03; 95% CI − 0.14 to 0.20, I^2^ = 76%). A possible explanation could be that the cohort studies included multiple confounding factors. The score in sample size ≤ 500 patients was significant in high but with moderate heterogeneity (MD 0.62; 95% CI 0.10 to 1.13, p = 0.003, I^2^ = 73%; Table 4; Fig. 3). In the six studies of subgroup of sample size ≤ 500 patients, there were three cross-sectional^7,17,18^ and three cohort studies^8,14,20^, the heterogeneity may be attributed to differences in study design. There was a nonsignificant difference in delay diagnosis over 5 years (MD 0.15; 95% CI − 0.22 to 0.51). BASDAI scores also had nonsignificant differences in the groups of diagnostic criteria of SpA, the sample size of patients > 500 patients and gender ratios (Table 4; Supplementary Fig. S4).

**Table 4:** Subgroup analysis of mean difference of BASDAI score in spondyloarthritis patients with and without uveitis of the included studies.

Subgroup	Mean difference	95% CI	*p* value	I^2^ (%)	n*
Delay diagnosis					
≤ 5 years	− 0.23	− 0.40 to − 0.07	0.005	0	3
> 5 years	0.15	− 0.22 to 0.51	0.43	67	4
Diagnostic criteria					
NY	0.38	− 0.15 to 0.92	0.16	36	3
ESSG	0.04	− 0.23 to 0.31	0.79	31	4
Study design					
Cross-sectional	0.69	0.23 to 1.16	0.003	0	3
Cohort	0.03	− 0.14 to 0.20	0.72	76	10
Sample size					
≤ 500 patients	0.62	0.10 to 1.13	0.02	73	6
> 500 patients	− 0.05	− 0.21 to 0.10	0.50	63	7
% male					
> 70%	0.33	− 0.14 to 0.79	0.17	64	4
≤ 70%	0.05	− 0.16 to 0.26	0.63	79	8

*Number of study.

### Publication bias

**Figure 4:**
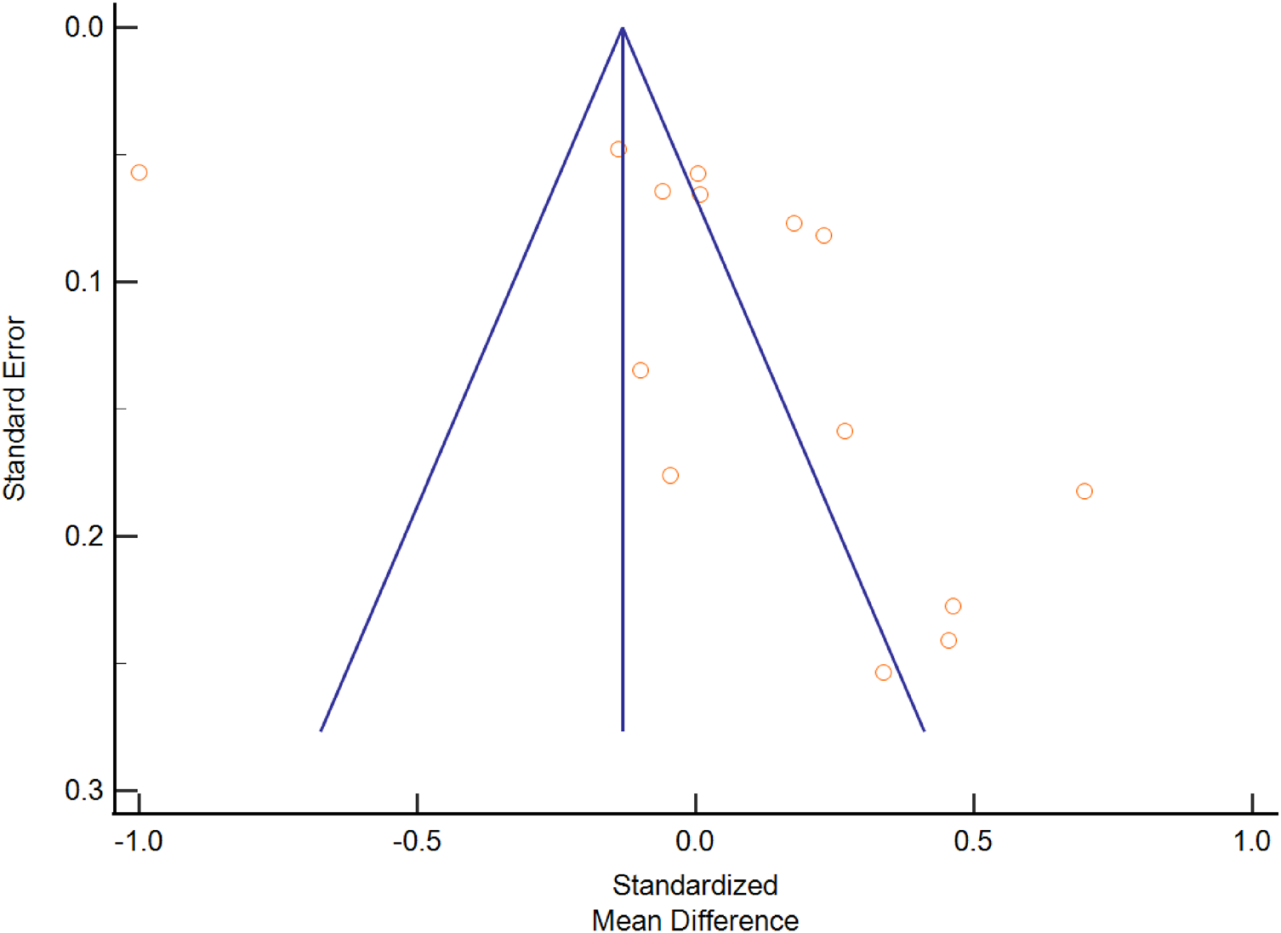
Funnel plot for publication bias.

The asymmetrical funnel plot indicated the publication bias in this meta-analysis (Fig. 4). We performed Egger’s test to confirm the existence of bias but without significance (t value = 4.25, df = 13, *p* = 0.14).

## Discussion

In a previous meta-analysis, the mean delay in the diagnosis of SpA was 6.7 (95% CI 6.2 to 7.2) years^11^, and in the present study, that of SpA with and without uveitis was 6.08 and 5.41 years, respectively. The delay in the diagnosis of SpA significantly differed between patients with and without uveitis (Table 3).

Uveitis can be the first presenting symptom of SpA, and studies have reported a prevalence of 40–50% of previously undiagnosed SpA in patients presenting with uveitis^3,13^. Wendling et al.^6^ reported that uveitis occurred before the first symptom of inflammatory back pain in 37% and simultaneously in 18% of cases. The delay in the diagnosis of SpA is associated with poor functional impairment, rapid radiographic progression, poor quality of life, and decreased treatment response^11^. In the present study, the pooled mean BASDAI score was the highest for a delay of 2–5 years (5.60, 95% CI 5.47 to 5.73). By contrast, the pooled mean BASFI score was the lowest for a delay of < 2 years (2.92, 95% CI 2.48 to 3.37), and its severity gradually increased to a delay of > 10 years (4.17, 95% CI 2.93 to 5.41; Table 3). Early recognition can lead to the early initiation of the most appropriate and effective therapy. Early diagnosis of SpA may reduce the adverse effects of the disease and improve functional ability. In this study, we found that patients with SpA with uveitis had a prolonged delay in diagnosis, which was highly associated with higher BASFI scores and poorer prognostic outcomes (Table 3). Awareness regarding uveitis is crucial considering its role in the diagnostic process of SpA for treatment choices and health-related quality of life^4^.

Backache or arthralgia were not the primary symptom of patients with SpA with uveitis during their presentation to an ophthalmologist. The degree of pain and disability, as reflected by BASDAI and BASFI scores, was often noted to be mild^13^. In the present study, the BASDAI score was significant lower in patients of SpA with uveitis with delay diagnosis less than 5 years (MD − 0.23; 95% CI − 0.40 to − 0.07). Previous studies have examined disease activity by using different assessment systems. Approximately 40–65% of patients with low BASDAI scores (< 4) had high disease activity, as measured using the ASDAS (≥ 2.1)^25–27^. In this study, we found a higher disease activity, as measured using the ASDAS (MD 0.18; 95% CI 0.11 to 0.25), in patients with SpA with uveitis than in those without. Measuring the BASDAI rather than the ASDAS is routine in clinical practice. However, some patients of uveitis especially early undiagnosed SpA suspected to have high disease activity despite low BASDAI scores should be examined using the ASDAS.

In the present study, we found no significant difference in the ESR between patients with and without uveitis (MD − 0.80; 95% CI − 4.62 to 3.03 mm/h). However, the CRP level tended to be higher in patients with uveitis (MD 1.48; 95% CI − 0.17 to 3.14 mg/L). Furthermore, we performed a subgroup analysis of ASDAS-CRP and ASDAS-ESR. Although the ASDAS might be affected by infectious or inflammatory conditions, it is regarded as more objective than the BASDAI. The ASDAS-CRP was significantly higher in patients with SpA with uveitis (MD 0.17; 95% CI 0.10 to 0.24). However, ASDAS-ESR did not differ between patients with and without uveitis (MD 0.03; 95% CI − 0.65 to 0.72; Table 3).

Uveitis may be a key feature leading to SpA diagnosis^6,12,17^. The nonspecific and often subtle symptoms of inflammatory back pain make early diagnosis and subsequent treatment challenging. In the subgroup analysis, the BASDAI score was significant higher in SpA patients with uveitis in study design as cross-sectional method (MD, 0.69; 95% CI 0.23 to 1.16). Patients with SpA with uveitis exhibited a marked reduction in the activities of daily living compared with those without uveitis (47.1% vs. 23.5%)^17,28^. However, in this study, no significant difference in BASFI scores was noted (MD − 0.12; 95% CI − 0.46 to 0.21). Because the delay in diagnosis was significantly longer in patients with uveitis, we further analyzed the pooled mean BASFI score. The lowest in delay diagnosis of < 2 years was 2.92 (95% CI 2.48 to 3.37), and the scores gradually increased to delay in diagnosis of > 10 years (4.17, 95% CI 2.93 to 5.41). Prolonged delay in diagnosis is common among patients with SpA and the occurrence of uveitis may be the reason for their first interaction with medical care, the occurrence of uveitis presents a unique opportunity for identifying such patients with undiagnosed SpA^29^. Therefore, awareness regarding uveitis among ophthalmologists and primary care physicians is vital considering its role in the diagnostic process and the selection of appropriate treatment for improving health-related quality of life^4^.

This study has some limitations that should be addressed. First, in our included studies, the disease activity indices of BASDAI, ASDAS and BASFI all require patients to answer the questionnaire and it is quite challenging for physician and patients to determine and make the assessment. Most of the SpA-related uveitis was diagnosed by ophthalmologists, however some of the history of uveitis was patients’ self-reported and this may reduce the accuracy. This may cause variations in findings among different geographical regions. Moreover, in terms of baseline characteristics, the included studies did not provide the age of uveitis diagnosis. We found the BASDAI was significant lower in patients of SpA with uveitis in delay diagnosis less than 5 years (MD − 0.23; 95% CI − 0.40 to − 0.07). Uveitis may occur before SpA symptoms or diagnosis^6,12,17^. The reason for a longer delay in diagnosis of SpA in patients with uveitis may be due to the mild symptoms of SpA. Additional cohort studies focusing on the age at diagnosis in patients of SpA and uveitis should be conducted.

In the present study, we found that patients with SpA with uveitis had a longer delay in diagnosis than did those without. Uveitis is the most frequent EAM in SpA^3^ and may affect disease activity, quality of life, and treatment decisions. In our meta-analysis, we found a significant difference in ASDAS-CRP and BASDAI between patients with and without uveitis. Screening patients of uveitis by using validated evaluation tool such as the DUET assessment algorithm^13^ allows for early identification of patients and the provision of an appropriate management plan. Early diagnosis of SpA may reduce the adverse effects of the disease and improve functional outcome of SpA patients.

## Methods

### Data source and search strategy

This systematic review and meta-analysis was performed according to the Preferred Reporting Items for Systematic Reviews and Meta-Analysis (PRISMA) guidelines^30^. The protocol for this review was registered in advance (PROSPERO: CRD42021285146). We performed a comprehensive search of peer-reviewed articles indexed in the electronic databases of PubMed, EMBASE, and Scopus. In addition, we examined the reference lists of the retrieved articles to identify additional studies for inclusion. The timeframe for the electronic search spanned from January 2000 to December 2021, and no language restriction was applied. Search terms were “ankylosing spondylitis”, “AS”, “spondylarthropathy”, “spondylarthritis”, “spondyloarthropathy”, “spondyloarthritis”, “SpA”, “uveitis”, “disease activity”, “BASDAI”, “BASFI”, and “ASDAS”. An extended search by using conference proceedings, conference abstracts, dissertations, and editorials was conducted to identify potentially relevant unpublished or gray literature.

### Study selection

Studies that met the following criteria were included in this study: (a) those including diagnostic criteria for SpA and AS, (b) those including patients diagnosed as having uveitis, and (c) those providing the mean value with the standard deviation (SD) of the BASDAI, BASFI, or ASDAS. We excluded review articles, case reports, and studies of duplicate populations.

Two authors (SL and CY) independently conducted the electronic search. Any disagreement was resolved by a third author (KY). When multiple articles were published for a single study, we used the most relevant publication and supplemented it with data from the authors’ other publications when necessary. The authors of studies were contacted when pertinent information was not available in the published version.

### Variables and outcome measures

The patients were diagnosed as having SpA according to the classification criteria of the European Spondylarthropathy Study Group^31^ and the Assessment of Spondyloarthritis International Society^32^. The diagnosis of AS was established according to the modified New York criteria^33^. PsA and SAPHO (synovitis, acne, pustulosis, hyperostosis, and osteitis) syndrome were diagnosed according to the Caspar classification criteria^34^ and Kahn criteria^35^, respectively.

The BASDAI consists of six numerical rating scales (scores ranging from 0 to 10) for measuring the severity of fatigue, spinal and peripheral joint pain, enthesitis, localized tenderness, and morning stiffness in patients with SpA^16^. The ASDAS includes the self-reported indices of back pain, duration of morning stiffness, peripheral joint pain and swelling, and patients’ global assessment of disease activity. In addition, the ASDAS includes laboratory measures, such as the CRP level and ESR, and each parameter is weighted and not simply added up as in the BASDAI^1,25^. The BASFI is used for assessing physical functioning in patients with SpA^36^.

### Quality assessment

We examined the risk of bias in the included studies according to the following six key domains by using the risk-of-bias assessment tool for nonrandomized studies (RoBANS)^24^: (a) selection of participants, (b) confounding variables, (c) measurement of exposure, (d) blinding of outcome assessments, (e) incomplete outcome data, (f) selective outcome reporting, and (g) other sources of bias. We graded each potential source of bias as yes, no, or unclear if the potential for bias was high, low, or unknown, respectively.

### Data synthesis and analysis

All statistical analyses were performed using Review Manager 5.4 (The Cochrane Collaboration, Oxford, UK) and MedCalc (Version 19.5.3, Ostend, Belgium)^37^. For continuous variables, mean differences (MDs) and 95% confidence intervals (CIs) were calculated. To perform a generic inverse variance meta-analysis, pooled mean values and 95% CIs were calculated from reported mean values with standard errors. When the mean delay to diagnosis was not reported, it was imputed as the difference in the mean age at symptom onset and the mean age at diagnosis. When the SD of diagnostic delay was missing, we imputed it by using methods recommended by Cochrane (based on the SD of age at onset, age at diagnosis, and their correlation in all studies)^38^.

Heterogeneity across studies was assessed through the I^2^ statistic. An I^2^ statistic of > 50% was considered to indicate substantial heterogeneity^39^. If substantial heterogeneity existed, subgroup analyses would be conducted to explore the heterogeneity. The primary analysis was based on published studies and unpublished conference proceedings. Because the meta-analysis included ≥ 10 studies, we investigated publication bias by using funnel plots^40^. A two-sided *p* value of ≤ 0.05 was considered statistically significant.

### Supplementary Information

Supplementary Figures.

## Data Availability

The authors confirm that the data supporting the findings of this study are available within the article and its supplementary materials.

## References

[1] van der Heijde D (2009). ASDAS, a highly discriminatory ASAS-endorsed disease activity score in patients with ankylosing spondylitis. Ann. Rheum. Dis..

[2] Braun J, Sieper J (2006). Early diagnosis of spondyloarthritis. Nat. Clin. Pract. Rheumatol..

[3] van Bentum RE, van der Horst-Bruinsma IE (2020). Axial spondyloarthritis in the era of precision medicine. Rheum. Dis. Clin. N. Am..

[4] Stolwijk C, van Tubergen A, Castillo-Ortiz JD, Boonen A (2015). Prevalence of extra-articular manifestations in patients with ankylosing spondylitis: A systematic review and meta-analysis. Ann. Rheum. Dis..

[5] Watad A, Bridgewood C, Russell T, Marzo-Ortega H, Cuthbert R, McGonagle D (2018). The early phases of ankylosing spondylitis: Emerging insights from clinical and basic science. Front. Immunol..

[6] Wendling D, Prati C, Demattei C, Miceli-Richard C, Daures JP, Dougados M (2012). Impact of uveitis on the phenotype of patients with recent inflammatory back pain: Data from a prospective multicenter French cohort. Arthritis Care Res..

[7] Berg IJ (2014). Uveitis is associated with hypertension and atherosclerosis in patients with ankylosing spondylitis: A cross-sectional study. Semin. Arthritis Rheum..

[8] Przepiera-Bedzak H, Fischer K, Brzosko M (2016). Extra-articular symptoms in constellation with selected serum cytokines and disease activity in spondyloarthritis. Mediators Inflamm..

[9] Man S (2021). Characteristics associated with the occurrence and development of acute anterior uveitis, inflammatory bowel disease, and psoriasis in patients with ankylosing spondylitis: Data from the Chinese ankylosing spondylitis prospective imaging cohort. Rheumatol. Ther..

[10] Lian F (2015). Anti-TNFalpha agents and methotrexate in spondyloarthritis related uveitis in a Chinese population. Clin. Rheumatol..

[11] Zhao SS, Pittam B, Harrison NL, Ahmed AE, Goodson NJ, Hughes DM (2021). Diagnostic delay in axial spondyloarthritis: A systematic review and meta-analysis. Rheumatology.

[12] Yasar BNS (2021). Uveitis-related factors in patients with spondyloarthritis: Treasure real-life results. Am. J. Ophthalmol..

[13] Haroon M, O'Rourke M, Ramasamy P, Murphy CC, FitzGerald O (2015). A novel evidence-based detection of undiagnosed spondyloarthritis in patients presenting with acute anterior uveitis: The DUET (Dublin Uveitis Evaluation Tool). Ann. Rheum. Dis..

[14] Essers I (2015). Characteristics associated with the presence and development of extra-articular manifestations in ankylosing spondylitis: 12-year results from OASIS. Rheumatology.

[15] Redeker I (2020). The impact of extra-musculoskeletal manifestations on disease activity, functional status, and treatment patterns in patients with axial spondyloarthritis: Results from a nationwide population-based study. Ther. Adv. Musculoskelet. Dis..

[16] Garrett S, Jenkinson T, Kennedy LG, Whitelock H, Gaisford P, Calin A (1994). A new approach to defining disease status in ankylosing spondylitis: The Bath Ankylosing Spondylitis Disease Activity Index. J. Rheumatol..

[17] Chen CH (2007). Association of acute anterior uveitis with disease activity, functional ability and physical mobility in patients with ankylosing spondylitis: A cross-sectional study of Chinese patients in Taiwan. Clin. Rheumatol..

[18] Gehlen M, Regis KC, Skare TL (2012). Demographic, clinical, laboratory and treatment characteristics of spondyloarthritis patients with and without acute anterior uveitis. Sao Paulo Med. J..

[19] Sampaio-Barros PD (2013). An analysis of 372 patients with anterior uveitis in a large Ibero-American cohort of spondyloarthritis: The RESPONDIA Group. Clin. Exp. Rheumatol..

[20] Diss JKJ, Georgiou A, Michael C, Rajan N, Shah A, Roussou E (2014). Differences in uveitis versus non-uveitis individuals with HLA-B27-postive spondyloarthritis with regard to first presenting symptoms. Rheumatology.

[21] da Costa IP (2015). Evaluation of performance of BASDAI (Bath Ankylosing Spondylitis Disease Activity Index) in a Brazilian cohort of 1,492 patients with spondyloarthritis: Data from the Brazilian Registry of Spondyloarthritides (RBE). Rev. Bras. Reumatol..

[22] Kasifoglu T (2018). Factors that may be associated with uveitis in patients with spondyloarthritis. Arthritis Rheumatol..

[23] Bilge, N. S. Y., Kasifoglu, T., & Kalyoncu, U. Uveitis related factors in patients with spondyloarthritis. In *EULAR Poster Presentations*, 491 (2019).

[24] Kim SY (2013). Testing a tool for assessing the risk of bias for nonrandomized studies showed moderate reliability and promising validity. J. Clin. Epidemiol..

[25] Nam B (2021). Low BASDAI score alone is not a good predictor of anti-tumor necrosis factor treatment efficacy in ankylosing spondylitis: A retrospective cohort study. BMC Musculoskelet. Disord..

[26] Fagerli KM (2012). Selecting patients with ankylosing spondylitis for TNF inhibitor therapy: Comparison of ASDAS and BASDAI eligibility criteria. Rheumatology.

[27] Marona J (2020). Eligibility criteria for biologic disease-modifying antirheumatic drugs in axial spondyloarthritis: Going beyond BASDAI. RMD Open.

[28] Gran JT, Skomsvoll JF (1997). The outcome of ankylosing spondylitis: A study of 100 patients. Br. J. Rheumatol..

[29] Khan MA, Haroon M, Rosenbaum JT (2015). Acute anterior uveitis and spondyloarthritis: More than meets the eye. Curr. Rheumatol. Rep..

[30] Moher D, Liberati A, Tetzlaff J, Altman DG, Group P (2009). Preferred reporting items for systematic reviews and meta-analyses: The PRISMA statement. PLoS Med..

[31] Dougados M (1991). The European Spondylarthropathy Study Group preliminary criteria for the classification of spondylarthropathy. Arthritis Rheum..

[32] Rudwaleit M (2011). The Assessment of SpondyloArthritis International Society classification criteria for peripheral spondyloarthritis and for spondyloarthritis in general. Ann. Rheum. Dis..

[33] van der Linden S, Valkenburg HA, Cats A (1984). Evaluation of diagnostic criteria for ankylosing spondylitis. A proposal for modification of the New York criteria. Arthritis Rheum..

[34] Taylor W (2006). Classification criteria for psoriatic arthritis: Development of new criteria from a large international study. Arthritis Rheum..

[35] Khan, M.A. The SAPHO-syndrome. In *Psoriatic Arthritis. Bailliere's Clinical Rheumatology* (H Wright Ed.), vol, 8 , 333–62 1994 (1994). 10.1016/s0950-3579(94)80022-7 8076391

[36] Calin A (1994). A new approach to defining functional ability in ankylosing spondylitis: The development of the Bath Ankylosing Spondylitis Functional Index. J. Rheumatol..

[37] Schoonjans F, Zalata A, Depuydt CE, Comhaire FH (1995). MedCalc: A new computer program for medical statistics. Comput. Methods Programs Biomed..

[38] Higgins, J. P. T. *et al*. Chapter 6, Section 6.5.2.8: Imputing standard deviations for changes from baseline. https://training.cochrane.org/handbook/current/chapter-06#section-6-5-2-8.

[39] Borenstein M, Borenstein M, Hedges LV, Higgins JPT, Rothstein S (2009). Fixed-effect versus random-effects models. Introduction to Meta-Analysis.

[40] Egger M, Davey SG, Schneider M, Minder C (1997). Bias in meta-analysis detected by a simple, graphical test. BMJ.

