# Supplementary material for: The effect of uveitis and undiagnosed spondyloarthritis: a systematic review and meta-analysis
Source: Sci Rep. 2023 Sep 7;13:14779. doi: 10.1038/s41598-023-41971-z (PMC10484908; doi:10.1038/s41598-023-41971-z)

**Supplementary Fig. 1 Risk of bias summary**


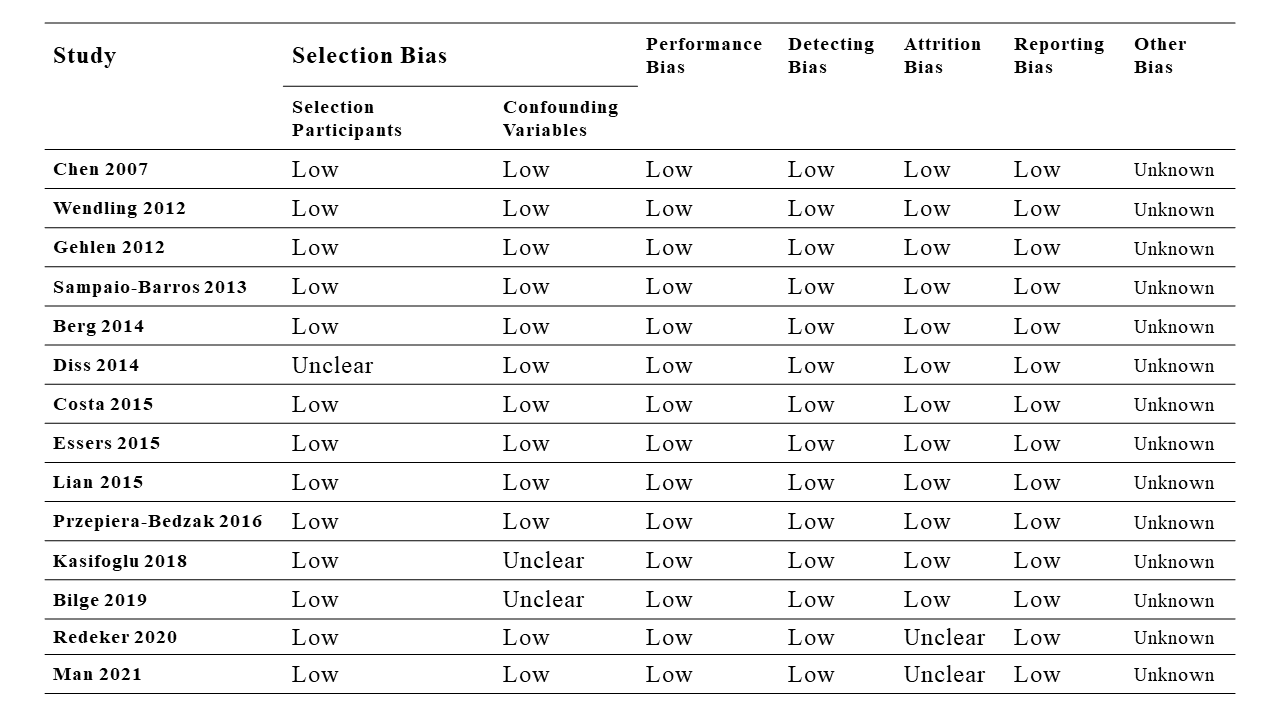


**Supplementary Fig. 2 Forest plot of the pooled mean of delay diagnosis, BASDAI, ASDAS, BASFI, CRP, ESR in patients of spondyloarthritis with and without uveitis**

1. Pooled mean of delay time of diagnosis in SpA with uveitis


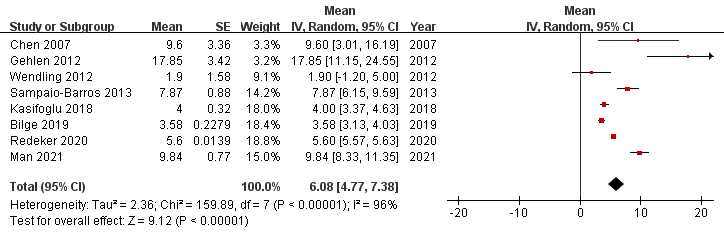


1. Pooled mean of delay time of diagnosis in SpA without uveitis


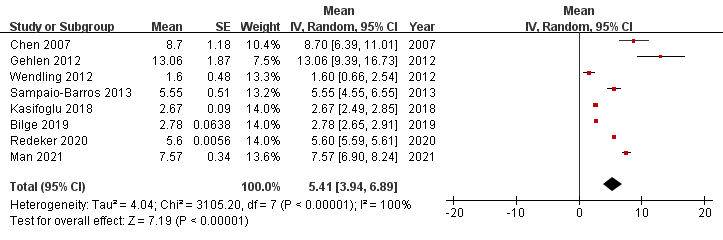


1. Pooled mean of ASDAS in SpA with uveitis


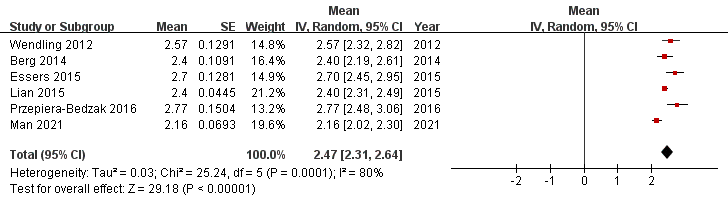


1. Pooled mean of ASDAS in SpA without uveitis


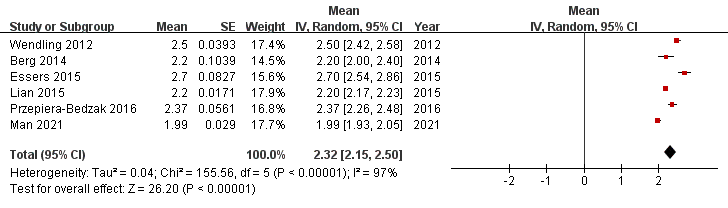


1. Pooled mean of BASDAI in SpA with uveitis


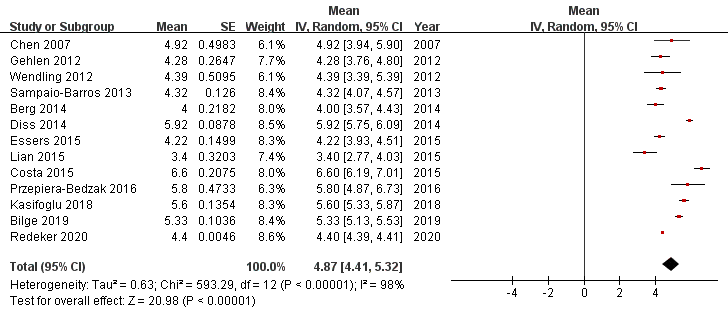


1. Pooled mean of BASDAI in SpA without uveitis


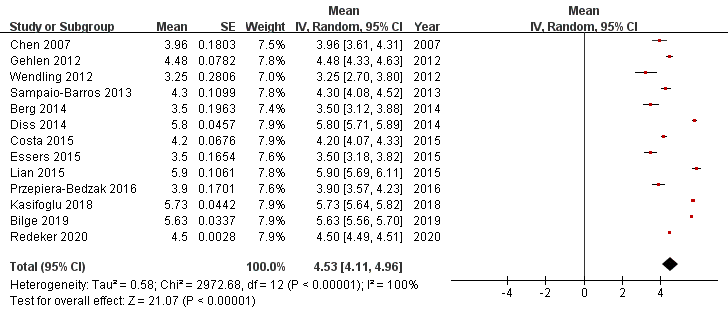


1. Pooled mean of BASFI in SpA with uveitis


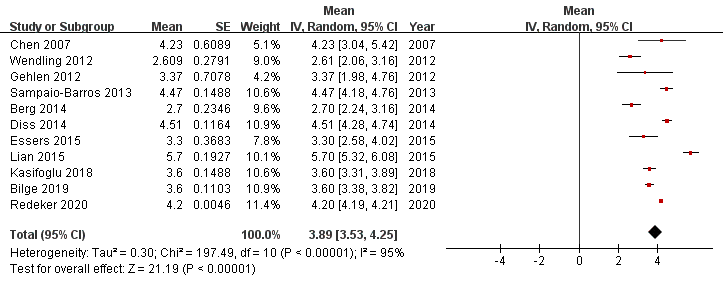


1. Pooled mean of BASFI in SpA without uveitis


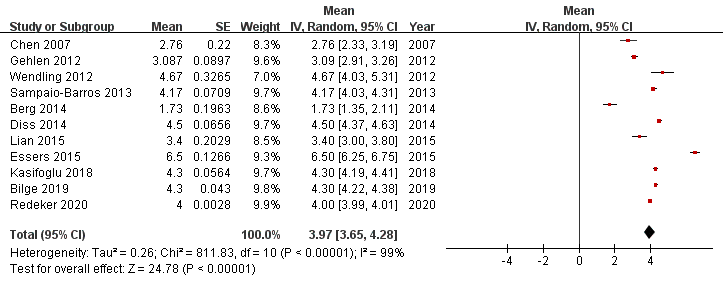


1. Pooled mean of CRP in SpA with uveitis


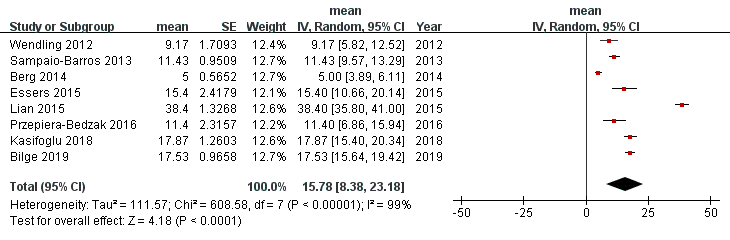


1. Pooled mean of CRP in SpA without uveitis


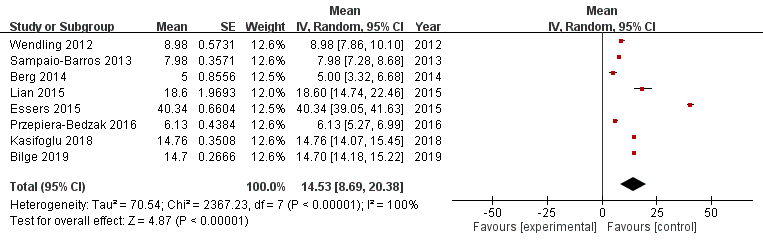


1. Pooled mean of ESR in SpA with uveitis


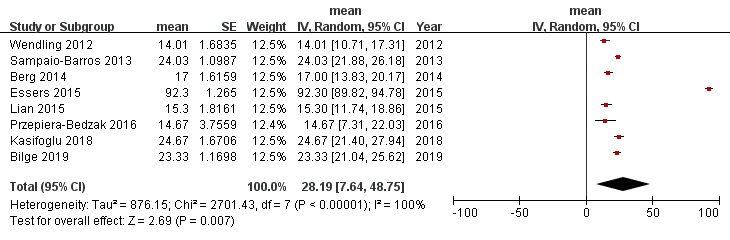


1. Pooled mean of ESR in SpA without uveitis


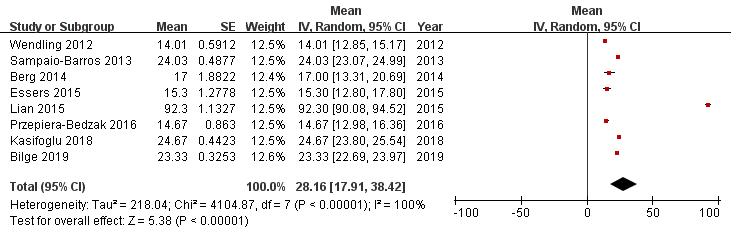


**Supplementary Fig. 3 Forest plot of the mean difference of ASDAS, ASDAS-ESR, BASFI, CRP, and ESR in patients of spondyloarthritis with and without uveitis**

ASDAS


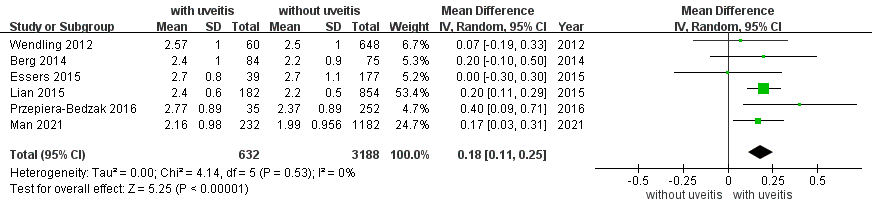


ASDAS-ESR


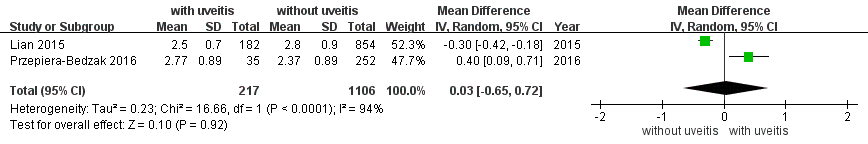


BASFI


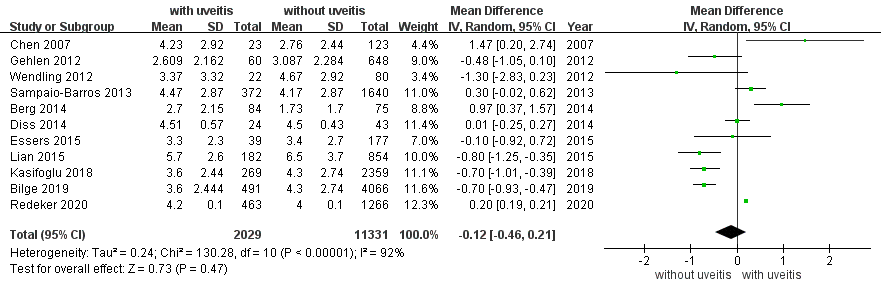


CRP


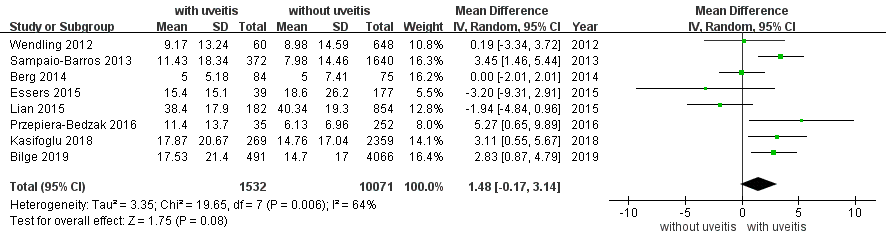


ESR


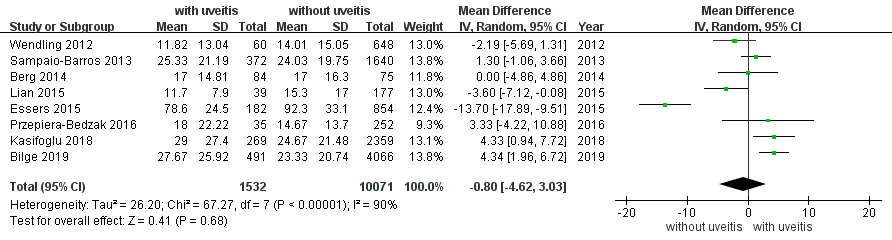


**Supplementary Fig. 4 Forest plot of the subgroup analysis of BASDAI in patients of spondyloarthritis with and without uveitis**

Delay Diagnosis over 5 years


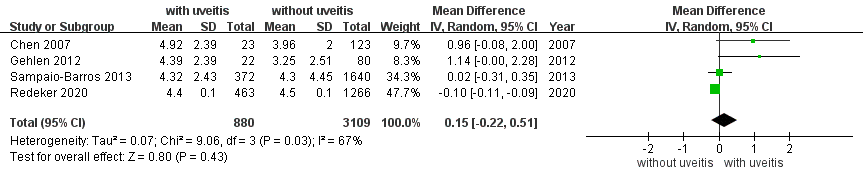


Study Design: Cohort


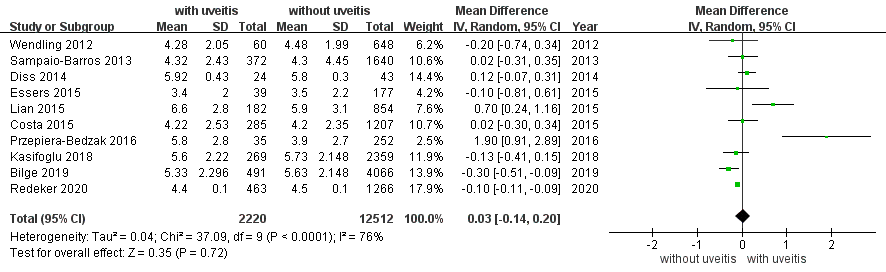


Diagnostic Criteria: NY


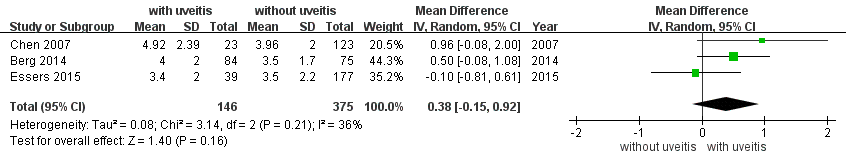


Diagnostic Criteria: ESSG


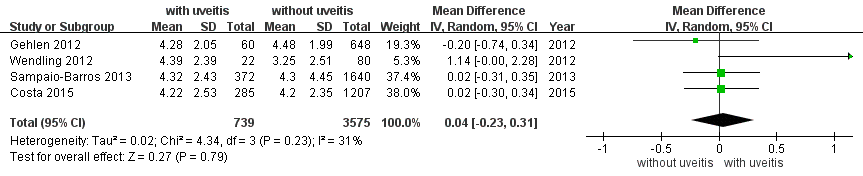


Sample Size >500 patients


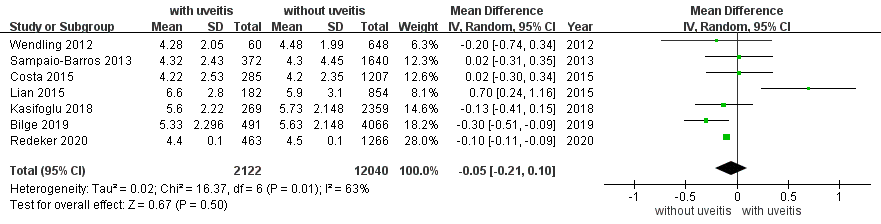


>70% male patients


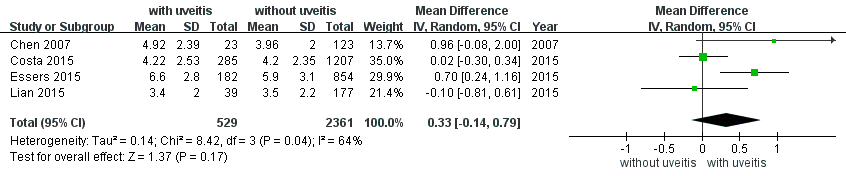


<70% male patients


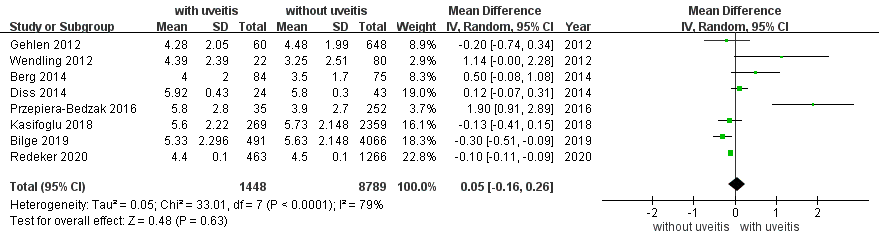

Supplement: Supplementary file 1 — Supplementary Figures. [file 41598_2023_41971_MOESM1_ESM.docx]
